# Homologous Proteins of the Manganese Transporter PAM71 Are Localized in the Golgi Apparatus and Endoplasmic Reticulum

**DOI:** 10.3390/plants9020239

**Published:** 2020-02-13

**Authors:** Natalie Hoecker, Anna Honke, Katharina Frey, Dario Leister, Anja Schneider

**Affiliations:** Molekularbiologie der Pflanze (Botanik), Department Biologie I, Ludwig-Maximilians-Universität München, 82152 Martinsried, Germany; Natalie.Hoecker@campus.lmu.de (N.H.); anna.honke@campus.lmu.de (A.H.); Ka.Frey@campus.lmu.de (K.F.); leister@lmu.de (D.L.)

**Keywords:** manganese transporter, UPF0016 protein family, PAM71, CMT1, Arabidopsis

## Abstract

Chloroplast manganese transporter 1 (CMT1) and photosynthesis-affected mutant 71 (PAM71) are two membrane proteins that function sequentially to mediate the passage of manganese across the chloroplast envelope and the thylakoid membrane. CMT1 and PAM71 belong to a small five-member protein family in *Arabidopsis thaliana*. The other three, photosynthesis-affected mutant 71 like 3 (PML3), PML4 and PML5 are not predicted to reside in chloroplast membranes. In this study, the subcellular localization of PML3:GFP, PML4:GFP and PML5:GFP was determined using transient and stable expression assays. PML3:GFP localizes to the Golgi apparatus, whereas PML4:GFP and PML5:GFP are found in the endoplasmic reticulum. We also examined patterns of *PML3*, *PML4* and *PML5* promoter activity. Although the precise expression pattern of each promoter was unique, all three genes were expressed in the leaf vasculature and in roots. Greenhouse grown single mutants *pml3*, *pml4*, *pml5* and the *pml4/pml5* double mutant did not exhibit growth defects, however an inspection of the root growth revealed a difference between *pml3* and the other genotypes, including wild-type, in 500 µM manganese growth conditions. Strikingly, overexpression of PML3 resulted in a stunted growth phenotype. Putative functions of PML3, PML4 and PML5 are discussed in light of what is known about PAM71 and CMT1.

## 1. Introduction

Plants, like all other living organisms, require manganese (Mn) as an activator of enzymes or as an integral component of protein complexes. The translocation of Mn from the soil into the aerial parts of plants requires the concerted action of a number of Mn transporters, which mediate uptake of Mn into root cells and facilitate subsequent Mn translocation from root to shoot [1,2]. At the cellular level, stably bound Mn is found in various locations, including the photosystem II (PSII) in chloroplasts, oxalate oxidase in cell walls, and Mn superoxide dismutase in mitochondria. Mn is also present in the Golgi apparatus (e.g., glycosyltransferases are activated by Mn^2+^), in the endoplasmic reticulum and in the vacuole. Indeed, the vacuole serves as an intracellular sink when Mn is in excess and as a source when the Mn supply is limited [3]. The most prominent role of Mn is its involvement in the oxygen-evolving complex of PSII, which splits water into oxygen, protons and electrons. The electrons released from water in PSII are eventually transferred to NADP^+^ via the cytochrome *b*_6_/*f* complex, plastocyanin, photosystem I and ferredoxin. Detailed analysis over the past several years has elucidated the structural basis for water oxidation and oxygen evolution in PSII by the catalytic Mn_4_CaO_5_ cluster [4,5,6,7].

To reach its destination in the oxygen-evolving complex of PSII in the thylakoid lumen, Mn must cross all three chloroplast membranes (the outer and inner envelopes and the thylakoid membrane). The outer envelope membrane is non-selectively permeable to most ions, while the inner envelope and the thylakoid membrane possess specific transport proteins. At the inner envelope membrane, chloroplast manganese transporter 1 (CMT1) represents the first candidate for the long-sought Mn import protein [8,9]. CMT1 transports Mn from the cytosol into the chloroplast stroma. The Arabidopsis mutant *cmt1* exhibits severe growth retardation, disruption of chloroplast ultrastructure and reduction of PSII activity, in association with a significant drop in the level of Mn in this organelle [8]. The protein responsible for the transport of Mn from the chloroplast stroma into the thylakoid lumen is photosynthesis-affected mutant 71 (PAM71) [10]. PAM71 resides in the thylakoid membrane and in *pam71* mutants PSII function is specifically impaired at the oxygen-evolving complex. The Mn transport function of CMT1 and PAM71 is conserved among homologous proteins from oxygenic photosynthetic organisms, e.g., cyanobacteria and green algae [10,11,12].

The presence of manganese in the Golgi apparatus, in the endoplasmic reticulum and in the vacuole means that transporter proteins of the endomembrane system and the tonoplast are important for the distribution of Mn in the cell [2]. Mn that is sequestered into the vacuole by members of the cation exchanger and calcium cation exchanger protein families is remobilized upon Mn deficiency by natural resistance-associated macrophage protein 3 (NRAMP3) and NRAMP4 in Arabidopsis mesophyll cells [2,13]. Another vacuolar Mn transporter, metal tolerance protein 8 (MTP8), participates in Mn homeostasis during seed development and germination in Arabidopsis plants [14,15]. A second member of the MTP family, MTP11, is found in Golgi-like and/or in pre-vacuolar compartments [16,17]. The *MTP11* gene is most highly expressed in leaf hydathodes and root tips and the protein could be involved in the exocytosis of excess Mn in these secretory tissues [16]. Two P-type ATPases, ER-type calcium ATPase 1 (ECA1) and ECA3 (which are localized to the endoplasmic reticulum and the Golgi membrane, respectively) are expressed in all major organs, and are involved in Mn pumping into these organelles [18,19,20]. Remobilization of Mn from the trans-Golgi compartment is mediated by NRAMP2 [21,22] which helps to build up/maintain the cytosolic Mn pool.

In addition to PAM71 and CMT1, the Arabidopsis genome encodes three other members of the uncharacterized protein family 0016 (UPF0016) [23], which we refer here as PML for PAM71-like proteins: PML3 (encoded by *At5g36290*), PML4 (encoded by *At1g25520)* and PML5 (encoded by *At1g68650*). According to the Transporter Classification Database (TCDB) [24] this protein family (classification number 2.A.106 in the TCDB) belongs to the LysE superfamily, this superfamily contains no other Mn transporters from plants. The three proteins PML3, PML4, and PML5 are predicted as substrates of the secretory pathway [10,23]. In the present study, we determined their subcellular localization and isolated insertion alleles. Moreover, we analyzed the spatial and temporal expression patterns by monitoring their activity of promoter fusions.

## 2. Results

### 2.1. PML3, PML4 and PML5 Are Predicted Substrates of the Secretory Pathway

**Figure 1:**
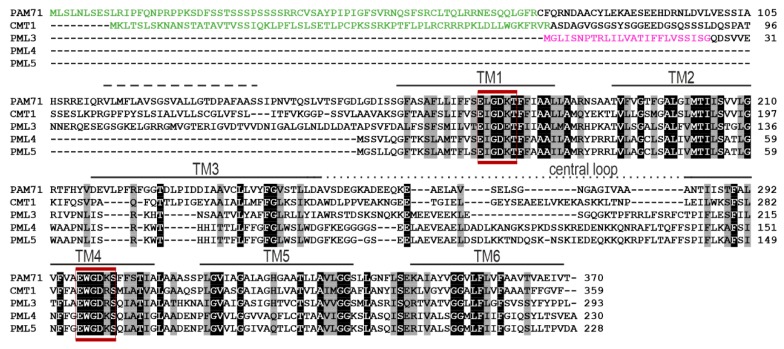
Protein sequence alignment of chloroplast manganese transporter 1 (CMT1), photosynthesis-affected mutant 71 (PAM71) and related proteins in Arabidopsis. Annotations were made as described previously [10]. Black and grey boxes indicate identical residues and conservative exchanges. Six putative transmembrane domains are indicated from TM1 to TM6, with the putative additional TM domain of CMT1 and PAM71 indicated by a dashed line. The conserved motifs E-x-G-D-(KR)-(TS) are highlighted by red lines, and the central loops are indicated by a dotted line. The putative chloroplast transit peptides are indicated by green letters and the putative secretory pathway transit peptide is indicated by magenta letters.

The UPF0016 family of membrane proteins comprises five members in Arabidopsis, and a sequence alignment is shown in Figure 1. PAM71 and CMT1 each contain a chloroplast-targeting signal (cTP) [8,10,23], whereas PML3 contains an N-terminal extension, which is predicted to be a secretory signal peptide (SP) [23] (Figure 1) and is notably lacking in PML4 and PML5 (Figure 1). The five proteins each possess six conserved transmembrane domains and share two highly conserved E-x-G-D-(KR)-(TS) motifs in transmembrane domain TM1 and TM4. In all five proteins, the central loop between transmembrane domain TM3 and TM4 is enriched in negatively charged acidic residues (Figure 1). Based on this sequence analysis, we concluded that PML3, PML4 and PML5 belong to the conserved group of Mn/Ca transporters, which are evolutionary conserved [23,25].

### 2.2. PML3 Localizes to the Golgi, and PML4 and PML5 Are Found in the Endoplasmic Reticulum

**Figure 2:**
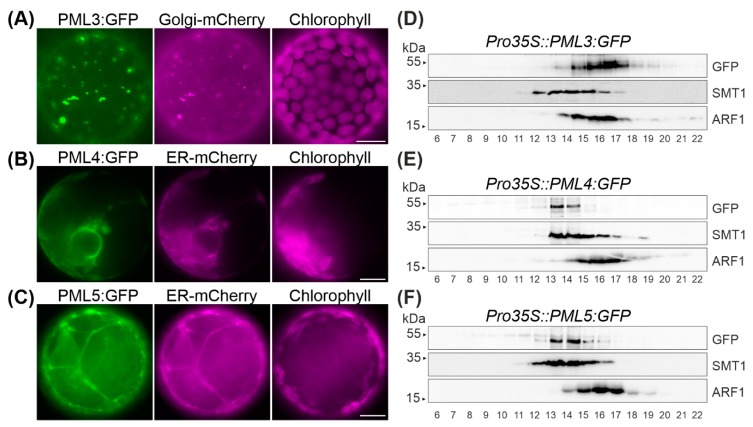
Fluorescence analysis of *Nicotiana benthamiana* protoplasts and immunoblot analysis of stable transformed Arabidopsis lines. (**A**) *N. benthamiana* leaves were infiltrated with pPro35S::PML3:GFP and pG-rk (=Golgi-mCherry); a second focus layer and corresponding overlay and bright field photographs are shown in Figure S1. (**B**) *N. benthamiana* leaves were infiltrated with pPro35S::PML4:GFP and pER-rk (=ER-mCherry); a second protoplast with overlay and bright field photographs is shown in Figure S1. (**C**) *N. benthamiana* leaves were infiltrated with pPro35S::PML5:GFP and pER-rk (=ER-mCherry). (**A**–**C**) GFP fluorescence of the indicated fusion protein is depicted in green, mCherry fluorescence and chlorophyll autofluorescence are depicted in magenta. Scale bar = 10 µm. (**D**–**F**) Separation of isolated microsomes of the indicated stable transformed Arabidopsis line by sucrose density centrifugation (20% to 50%). Top-down aliquots of fractions as indicated, were separated by SDS-PAGE and analyzed by immunodetection using anti-GFP, anti-sterol methyltransferase 1 (SMT1) and anti-ADP-ribosylation factor 1 (ARF1) antibodies. The individual lines used for this experiment were *Pro35S::PML3:GFP* #1, *Pro35S::PML4:GFP* #1 and *Pro35S::PML5:GFP* #1, as indicated in Figure S2.

In order to determine the subcellular localization of PML3, PML4 and PML5, the open reading frames of the respective cDNAs were amplified and subsequently cloned upstream of the *GFP* reporter gene and downstream of the 35S promotor. The resulting constructs were then transiently expressed in *Nicotiana benthamiana* leaves (Figure 2 and Figure S1). In none of these cases was the GFP signal found to overlap with chlorophyll autofluorescence (Figure 2). Thus, a chloroplast localization for PML3, PML4 and PML5 can be excluded, as already suggested by the in silico analysis (Figure 1). The pattern of PML3:GFP fluorescence displayed predominantly dot-like signals resembling those of the fluorescent Golgi marker GmMAN1:mCherry [26]. Indeed, when simultaneously expressed, fluorescence signals from both proteins PML3:GFP and GmMAN1:mCherry overlapped, thus indicating that PML3:GFP is targeted to the Golgi apparatus (Figure 2A, Figure S1A). We also determined the subcellular localization of PML4:GFP and PML5:GFP. The GFP fluorescence of both protein fusions was distributed in a pattern resembling that of the ER marker AtWAK2:mCherry [26] and coincided exactly with the red fluorescence of this marker (Figure 2B,C, Figure S1B), indicating that both proteins are targeted to the endoplasmic reticulum. We verified that the GFP fluorescence of PML5:GFP did not overlap with the red fluorescence of GmMAN:mCherry, effectively excluding the possibility of a dual localization (Figure S1C).

To further confirm these findings, we generated stably transformed Arabidopsis lines expressing *PML3:GFP*, *PML4:GFP* and *PML5:GFP* under the control of the 35S promoter and named these individual lines *Pro35S::PML3:GFP* #1, *Pro35S::PML4:GFP* #1 and *Pro35S::PML5:GFP* #1, respectively (Figure S2). Microsomal fractions from leaves of the three lines were subjected to centrifugation on sucrose-density gradients (Figure 2D–F). Fractions 6 to 22 were subjected to immunoblot analysis employing three antibodies, including the GFP antibody. Antibodies against sterol methyltransferase 1 (SMT1), an integral membrane protein specific to the endoplasmic reticulum [27], and ADP-ribosylation factor 1 (ARF1), a protein that is found in both the Golgi and the trans-Golgi network [28], were used to detect endogenous proteins of the respective compartment (Figure 2D,F). PML3:GFP was found in the same fractions as the ARF1 signal (Figure 2D), while the GFP fusions expressed in the lines *Pro35S::PML4:GFP* and *Pro35S::PML5:GFP* were both found to comigrate with the SMT1 protein (Figure 2E,F). These observations confirm the localization of PML3 to the Golgi apparatus and the assignments of PML4 and PML5 to the endoplasmic reticulum.

### 2.3. Transgenic Arabidopsis Lines Expressing PML3:GFP Show a Stunted Growth Phenotype

**Figure 3:**
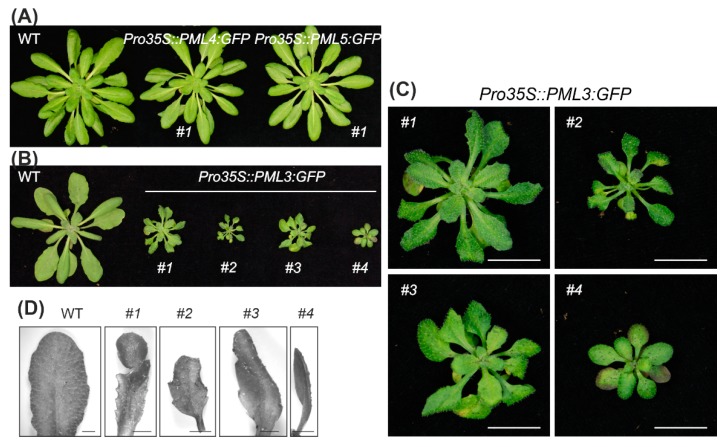
Photographs of stably transformed Arabidopsis lines. (**A**) *Pro35S::PML4:GFP* and *Pro35S::PML5:GFP* plants in comparison to a wild-type plant are shown. (**B**) WT and transgenic lines *Pro35S::PML3:GFP* #1, #2, #3, #4 were grown for 3 weeks under a 12 h/12 h light–dark cycle and 2 weeks in the greenhouse. (**C**) Magnified images of plants shown in (B), Scale bar = 1 cm. (**D**) Magnified images of individual leaves taken from the plants shown in (B) and (C), Scale bar = 2 mm.

The stably transformed Arabidopsis lines *Pro35S::PML4:GFP* #1 and *Pro35S::PML5:GFP* #1 possessed a wild-type-like phenotype (Figure 3A), however *Pro35S:PML3:GFP* #1 showed an interesting leaf phenotype. The rosette of *Pro35S::PML3:GFP* #1 was much smaller than a wild-type rosette of the same developmental stage (Figure 3B), and individual leaves exhibited a prominent constraint and bent morphology (Figure 3C,D). A rather simple explanation for this growth phenotype could be that the transgene caused a mutation by its random insertion into the genome of Arabidopsis. To exclude this possibility, we searched for independent transgenic *Pro35S::PML3:GFP* lines that exhibited GFP expression. To this end, we isolated three further lines, named *Pro35S::PML3:GFP* #2, #3, and #4 (Figure S2). All lines were grown at the same time and visually inspected for their leaf phenotype. It turned out that they were smaller than wild-type of the same age; thus, it is likely that expression of *PML3:GFP* evokes the stunted growth phenotype. Moreover, two lines, *Pro35S::PML3:GFP* #2 and #3, displayed a similar curvature of individual leaves in agreement with observations made in *Pro35S::PML3:GFP* #1 (Figure 3). It should be noted that not all leaves are equally affected and therefore it is maybe not surprising that *Pro35S::PML3:GFP* #4 did not show curvature of individual leaves (Figure 3).

### 2.4. Expression Patterns of PML3, PML4 and PML5 in Arabidopsis Tissues

**Figure 4:**
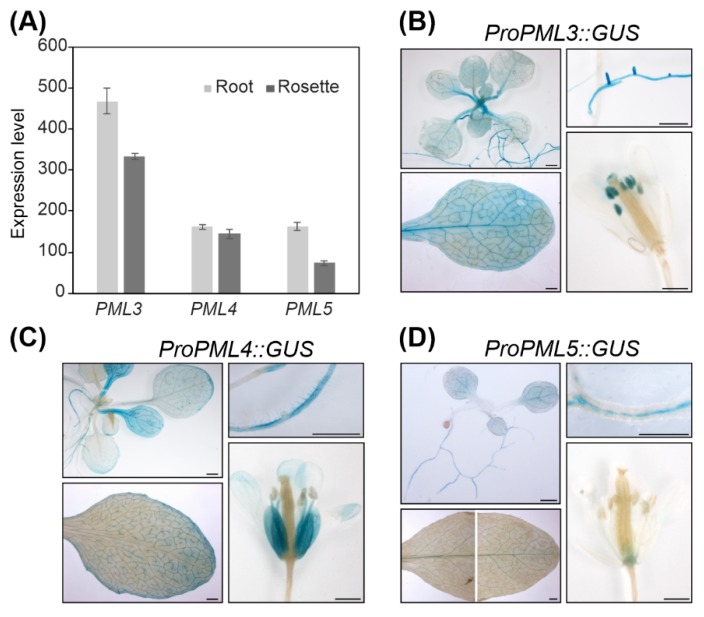
Analysis of *PML3*, *PML4*, and *PML5* expression. (**A**) Expression levels were derived from the developmental map of the Arabidopsis Browser Database [29]. (For promotor analysis, various tissues of Arabidopsis line *ProPML3::GUS* (**B**), *ProPML4::GUS* (**C**), and *ProPML5::GUS* (**D**) were incubated in staining solution and analyzed for a blue precipitate of chloro-bromoindigo indicating β-Glucuronidase (GUS) activity. Please note that the leaf photograph in (D) is composed of two single photographs. Scale bar = 1 mm.

From the results so far, we concluded that PML4 and PML5 localize to the same compartment and thus might have redundant functions in the cell. The expression pattern revealed that *PML4* and *PML5*, as well as *PML3*, are expressed in photosynthetic and in non-photosynthetic tissues (Figure 4A and Figure S3). To define the expression patterns more specifically, promoter-driven reporter gene constructs were assembled by fusing a 1.3-kb fragment upstream of the *PML3* coding sequence and 1.2-kb segments of the upstream regions of *PML4* and *PML5*, respectively, to the *uidA* reporter gene. The constructs were designed to exclude the ATG of the endogenous genes; more precisely, the PML3 fragment comprises −1021 bp to +315 bp from the transcription initiation site, the PML4 fragment comprises −971 bp to +237 bp from the transcription initiation site, and the PML5 fragment comprises −1116 bp to +94 bp from the transcription initiation site (sequences of the fragments are shown in Table S1). Transgenic lines harboring the individual constructs were generated and named *ProPML3::GUS*, *ProPML4::GUS* and *ProPML5::GUS*. We found β-Glucuronidase (GUS) activity in rosette leaves and in roots of the transgenic line *ProPML3::GUS*, *PML3* promoter-driven GUS expression was strong in lateral roots, throughout the leaf and particularly in the vasculature and in anthers (Figure 4B). Both *ProPML4::GUS* and *ProPML5::GUS* lines showed similar yet distinct GUS activity patterns. In both lines, GUS activity was found in roots and leaves; however, *PML4*-driven GUS expression was found mainly in minor veins, and *PML5*-driven GUS expression was predominantly detected in the main veins of adult leaves (Figure 4C,D). The *ProPML4::GUS* line expressed GUS particularly in root hairs, while in line *ProPML5::GUS*, the GUS activity was found in the root stele (Figure 4C,D). Moreover, the patterns of *PML4* and *PML5* promoter expression differed from each other in flower tissues (Figure 4C,D). Only weak GUS activity in the receptacle tissue of *ProPML5::GUS* was detected, whereas petals of *ProPML4::GUS* displayed strong GUS activity. Taken together, this analysis shows that *PML4* and *PML5* are expressed in distinct tissues of roots, leaves and flowers, which suggests that they might have partially redundant functions in plants.

### 2.5. Root Elongation in pml3 Is Distinct from Other Genotypes in 500 µM MnSO_4_

**Figure 5:**
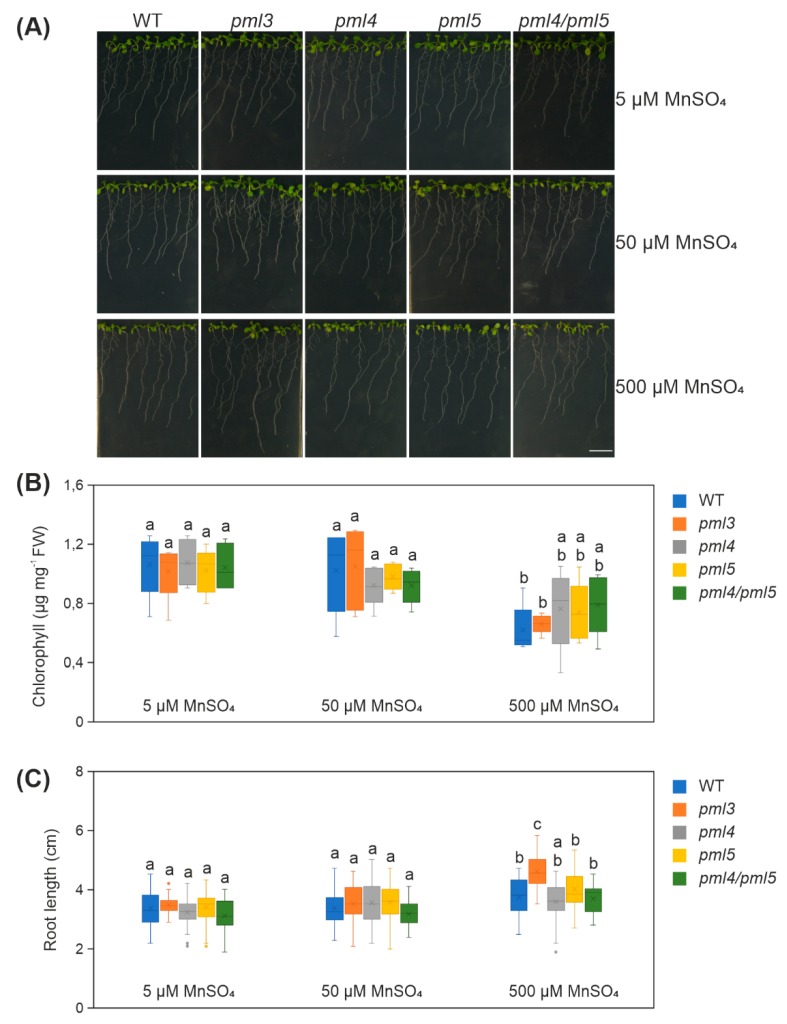
Analysis of wild-type, *pml3*, *pml4*, *pml5* and *pml4/pml5* grown under different Mn regimes (**A**) Photographs were taken from representative samples. Plants were grown in the indicated Mn condition, in a vertical position under a 16 h/8 h light–dark cycle at 100 µmol photons m^−2^ s^−1^ for 10 days. Scale bar = 1 cm (**B**) Five to six plants per genotype and condition were combined for one chlorophyll extraction and at least five extractions were prepared. Data are depicted as boxplots representing the range of values, the exclusive median and the mean, indicated as x (*n* ≥ 5). FW = fresh weight. (**C**) The length of the primary root of all plants are depicted as boxplots representing the range of values, the exclusive median and mean, indicated as x (*n* ≥ 35 per genotype and condition). Outliers are indicated as dots. (**B**,**C**): different letters indicate statistical significance according to ANOVA (*p* < 0.05, Tukey’s honestly significant difference (HSD) test). Mean values and standard deviations are given in Tables S2 and S3. Poorly germinated seeds were excluded from the analysis.

We isolated the insertion alleles *pml3*, *pml4* and *pml5* (Figure S4), and named the mutant lines accordingly. From our observations so far, we also aimed to generate a double mutant *pml4/pml5* to test whether PML4 and PML5 have a redundant function. Single and double mutant plants grown in the greenhouse did not display differences compared to wild-type plants (Figure S4). Thus, we examined the mutants at an earlier stage, with a focus on roots, because *PML3*, *PML4* and *PML5* expression is higher in roots than in rosette leaves (Figure S3). Seeds of wild-type, *pml3*, *pml4*, *pml5,* and *pml4/pml5* were sterilized and grown for ten days to inspect for a rosette-leaf phenotype and for root phenotype. The five genotypes were grown on medium containing 5 µM MnSO_4_, 50 µM MnSO_4_, and 500 µM MnSO_4_ (Figure 5A).

As a read-out for a rosette-leaf phenotype, we determined chlorophyll content. The chlorophyll content of the five genotypes is subjected to little fluctuation when plants were grown in 5 µM and 50 µM MnSO_4_, indicating that Arabidopsis can grow well within a certain range of Mn. The overall chlorophyll content went down when plants were subjected to 500 µM MnSO_4_ and a comparison between the five genotypes revealed that they behave similarly (Figure 5B, Table S2). As a read-out for a root phenotype, we determined the length of the primary root and observed that this parameter is also subjected to little fluctuation when plants were grown in 5 µM and 50 µM MnSO_4_ (Figure 5A,C). The overall root length increased when plants were subjected to 500 µM MnSO_4_ (Figure 5C); in another study, this tendency was observed when Arabidopsis plants were treated with 1250 µM MnSO_4_ [30]. Interestingly, a comparison of the root length revealed that roots of *pml3* grown in 500 µM MnSO_4_ were significantly more elongated than roots of wild-type, *pml4, pml5* and *pml4/pml5* (Figure 5C, Table S3) in 500 µM MnSO_4_ and in any other condition. To investigate whether this effect can be inverted, plants were grown on medium with the MnSO_4_ concentration set to 50 nM (Figure S5). Plant growth was not drastically impaired, indicating that internal Mn stores possibly support plant growth to a certain degree. The overall root length decreased slightly in this condition (Table S3), in agreement with a trend described by Gruber et al. [31]. However, we could not detect any difference between the genotypes regarding root length and chlorophyll content (Figure S5). Taken together, we concluded that the 500 µM MnSO_4_ treatment caused an intracellular excess of Mn which influences root elongation and that PML3 is involved in this process. Consistent with other results, this finding indicates that the functionality of PML3 is distinct from that of PML4 and PML5.

## 3. Discussion

The genome of Arabidopsis contains three sequences that code for proteins with high similarity to PAM71 and CMT1, two previously characterized Mn transporters localized in distinct chloroplast membranes [8,9,10]. Like PAM71 and CMT1, the three predicted protein sequences contain the two highly conserved E-x-G-D-(KR)-(TS) motifs, with two negatively charged acidic residues in TM1 and TM4, which provide a suitable environment for the passage of Mn^2+^ ions and maybe other cations through membranes. The greatest diversity within this protein family is found in their N-terminal regions (Figure 1), which were proposed to be responsible for targeting the respective protein to the correct membrane [23]. In the present study, we were able to localize PML3, PML4 and PML5 in the endomembrane system of Arabidopsis.

The PML3 protein sequence contains an N-terminal region predicted to serve as signal peptide for the secretory pathway (Figure 1), and indeed PML3 could be localized to the Golgi apparatus (Figure 2, Figure S1), most likely in the Golgi membrane. PML3, together with its homologs, belongs to the UPF0016 family of membrane proteins, and the yeast and human members of this family were found to be located in the Golgi membrane as well [32]. Recent findings support a role for the human member transmembrane protein 165 (TMEM165) in Ca^2+^/Mn^2+^ import into the Golgi. Indeed, Mn^2+^ is known to be an essential cofactor for many Golgi-localized glycosyltransferases [33] and glycosylation abnormalities have been described in humans with mutations in TMEM165, which result in a rare genetic condition called Congenital Disorder(s) of Glycosylation [34]. Plant glycosyltransferases with functions in glycoprotein biosynthesis are also present in the Golgi [35,36]. In addition, glycosyltransferases in plants are involved in the biosynthesis of non-cellulosic polysaccharides of the cell wall [35], and some of them have been shown to depend on Mn^2+^ [37]. It is perhaps not surprising that the *pml3* insertion line does not show a drastic altered phenotype compared to wild-type (Figure 5, Figure S4), as at least one other protein, the P-type ATPase ECA3, is known to be located in the Golgi membrane and possibly plays a role in the transport of Mn into this organelle [19]. *ECA3* is expressed in all major organs, and particularly in the vasculature of primary and lateral roots, as well as in leaves and flowers [19,20]. Its expression thus overlaps with *PML3* expression, which was found in all major organs, especially in the vasculature of leaves and in roots (Figure 4). The growth of the *eca3* mutant is impaired when grown in Mn deficiency [19,20], whereas in *pml3,* only a subtle root phenotype appeared (Figure 5). It appears that primary root elongation in *pml3* plants is induced more in 500 µM MnSO_4_ conditions (Figure 5). Because the root length of *pml3* plants was not changed in comparison to wild-type in lower MnSO_4_ concentrations, we assume that PML3 is involved in specific processes during Mn excess, perhaps loading Mn into the Golgi. It is believed that Mn stored in the Golgi can be remobilized by NRAMP2 in Mn deficient conditions [21,22]. In addition, an interesting phenotype was observed when *PML3:GFP* was overexpressed. A stunted growth phenotype occurred in *PML3:GFP* over-expressor lines (Figure 3), with some leaves being out of shape. The underlying molecular mechanism evoking this phenotype is unknown, however one speculation is that massive Mn content in the Golgi apparatus of the over-expressor lines might alter glycosylation reactions, which eventually affects leaf shape and plant growth. Although highly speculative, this hypothesis certainly deserves deeper investigation in the future.

The subcellular localization of PML4 and PML5 was also determined and, unlike PML3, both proteins were found to reside in the endoplasmic reticulum (Figure 2, Figure S1). The importance of the endoplasmic reticulum in N-glycosylation of glycoproteins and in the biosynthesis of glycans for the cell wall matrix is well known [38,39]. Furthermore, the peptidyl serine O-α-galactosyltransferase (SGT1), which is involved in *O*-glycosylation, localizes to the endoplasmic reticulum, and was shown to require Mn^2+^ as a cofactor [40]. Mn transport into the endoplasmic reticulum is mediated by ECA1, and the *eca1* mutant showed increased sensitivity towards high Mn; in fact, it failed to elongate the root hairs under these conditions, presumably through impairment in growth tip processes [18]. We propose that PML4 and PML5 might act to fine tune Mn allocation into the endoplasmic reticulum of specific cell types, e.g., those of the root hair (PML4) or root stele (PML5) (Figure 4), as they are dispensable for primary metabolism. The interplay of various Mn transporters within single cells as well as on different tissue levels is complicated by the fact that some of them also transport other cations like calcium, or indirectly act on calcium homeostasis. Thus, the challenge of future work will be to elucidate the precise substrate specificity of PML3, PML4 and PML5 and perhaps other Mn transporters by employing heterologous expression and reconstitution assays. Furthermore, it will be interesting to learn more about the sophisticated network of Mn transporters, perhaps through the generation of triple or even higher order mutant lines.

## 4. Materials and Methods

### 4.1. Plasmid Construction and Plant Material

For subcellular localization studies, the sequences encoding PML4 and PML5 were amplified using specific primer combinations (Table S4) and single-stranded Arabidopsis cDNAs. The amplified cDNAs were ligated into pENTR-TOPO (Life Technologies, Carlsbad, CA, USA), and recombined into pB7FWG2 [41], yielding the plasmids pPro35S::PML4:GFP and pPro35S::PML5:GFP. The plasmid pPro35S::PML3:GFP was constructed from appropriate primers (Table S4), the backbone of pB7FWG2 and a template cDNA using the Gibson Assembly Cloning Kit (catalog number E5510S, New England Biolabs, Ipswich, MA, USA) in accordance with the manufacturer’s instructions.

For study promoter activities, fragments located upstream of the *PML3, PML4* and *PML5* coding regions (Table S3) were amplified from Arabidopsis genomic DNA using specific primer combinations (Table S4) and ligated into pENTR-TOPO. The entry clones were subsequently recombined into pKGWFS7 [41] upstream of the *uidA* reporter gene, yielding the plasmids pProPML3::GUS, pProPML4::GUS and pProPML5::GUS. Thus, the constructs were designed to use the ATG of the *uidA* reporter gene.

All plasmids were transformed into *Agrobacterium tumefaciens* strain GV3101. Agrobacterium strains harboring pPro35S::PML3:GFP, pPro35S::PML4:GFP or pPro35S::PML5:GFP were either infiltrated into *N. benthamiana* leaves or used for stable transformation of Arabidopsis by the floral dip method. Individual transgenic Arabidopsis plants were selected on the basis of their resistance to ammonium glufosinate (100 mg L^−1^). Agrobacterium strains harboring pProPML3::GUS, pProPML4::GUS or pProPML5::GUS were also used for stable transformation of Arabidopsis. In this case, individual transgenic Arabidopsis plants were selected on the basis of their resistance to kanamycin (50 µg mL^−1^).

Arabidopsis mutant lines were obtained from the European Arabidopsis Stock Centre (NASC) and named *pml3* (N402563=GK-027F07), *pml4* (N664220=SALK_143524) and *pml5* (N438509=GK-402B01), respectively. Genotyping of all lines was performed using PCR with appropriate primer combinations (Table S3). Expression of *PML3*, *PML4* and *PML5* in the different genotypes was analyzed by RT-PCR using an appropriate primer combination (Table S4), first strand cDNA and 30 PCR cycles.

Arabidopsis wild-type plants (accession Columbia-0) were used for stable transformation by the floral dip method [42] and as controls. Unless stated otherwise, Arabidopsis lines and *N. benthamiana* were cultivated in a temperature-controlled greenhouse with additional lighting up to 16 h to reach at least 140 µmol photons m^−2^ s^−1^. Surface-sterilized Arabidopsis seeds were germinated on half-strength Murashige and Skoog medium (½ MS) including 1% (*w*/*v*) sucrose, with or without antibiotic, and grown for two weeks before individual plants were transferred to the greenhouse. For growth on media with different Mn concentrations, the macro and micro elements of ½ MS were mixed according to the Murashige and Skoog medium composition (Duchefa M0222), with the exception of MnSO_4_: 0.00845 mg L^−1^, 0.845 mg L^−1^, 8.45 mg L^−1^ or 84.5 mg L^−1^ of MnSO_4_ × H_2_O were added in 1 L of medium to give a final concentration of 50 nM, 5 µM, 50 µM or 500 µM MnSO_4_, respectively. Plant agar (Duchefa P1001) was used for solidification.

### 4.2. Protoplast Isolation and Fluorescence Microscopy

Agrobacterium-mediated infiltration of *N. benthamiana* was performed as described [43]. Protoplasts were isolated from leaf tissue 48 h after infiltration [43] and fluorescent signals were detected using an Axio-Imager fluorescence microscope (Carl Zeiss, Oberkochen, Germany). Two plasmids expressing marker proteins for either the Golgi (pG-rk expressing GmMAN1:mCherry) or the endoplasmic reticulum (pER-rk expressing AtWAK2:mCherry) were chosen for co-infiltration, because their specificity is well established [26,44]. The GFP fluorescence was excited at 470 ± 40 nm and the emission recorded at 525 ± 50 nm. Chlorophyll autofluorescence was excited at 450–490 nm, and emission was recorded at >515 nm. The mCherry fluorescence of the marker proteins was excited at 560 ± 40 nm and the emission recorded at 630 ± 75 nm. The fluorescence signals obtained in the different channels were overlaid using ImageJ (version 1.51j).

### 4.3. Microsomal Preparation and Western Blot Analysis

For the isolation of microsomal fractions, transgenic Arabidopsis lines were grown for 4–5 weeks in a growth chamber under a 12 h/12 h light–dark cycle at 100 µmol photons m^−2^ s^−1^ and 22 °C/18 °C. Leaf homogenization and microsomal fraction enrichment by differential centrifugation was performed as previously described [45]. Isolated microsomal fractions were layered on top of a continuous 20–50% (*w*/*v*) sucrose density gradient and centrifuged for 16 h at 4 °C and 100,000× *g*. After centrifugation, 500 µL fractions were collected form the top of the tube and analyzed by SDS-PAGE and Western blotting [45]. Individual proteins were detected using antisera raised against GFP (Life Technologies A6455, 1:2000 dilution), SMT1 (Agrisera AS07 266, Vannas, Sweden; 1:500 dilution) and ARF1 (Agrisera AS08 325, 1:1000 dilution) in combination with anti-rabbit IgG horseradish peroxidase (HRP) (Sigma Aldrich, St. Louis, MO, USA 1:25,000 dilution) and the Pierce Enhanced Chemiluminescence System (Thermo Fisher Scientific, Waltham, MA, USA).

### 4.4. Promotor Activity Analysis and Light Microscopy

To monitor tissue-specific promoter activity, transgenic Arabidopsis lines were assayed for β-glucuronidase (GUS) activity. After overnight incubation in staining solution (100 mM NaH_2_PO_4_ pH 7.0, 10 mM ethylenediaminetetraacetic acid (EDTA), 0.1% (*v*/*v*) Triton X-100, and 0.5 mg mL^−1^ 5-bromo-4-chloro-3-indolyl glucuronide (X-Gluc)) at 37 °C, tissues were cleared in 70% (*v*/*v*) ethanol and analyzed with a stereoscope (SteREO Lumar.V12, Carl Zeiss) equipped with an AxioCam color camera.

### 4.5. Real-time PCR Analysis

Total RNA was isolated from the root and shoot systems of Arabidopsis wild-type plants using TRIzol reagent (InVitrogen). For real time qRT-PCR, SYBR Green Supermix (Bio-Rad) was used, and PCR was performed with the iQ5 multi-color real-time PCR detection system (Bio-Rad). Quantification of relative expression levels was performed using the comparative cycle threshold (C_T_) method [46].

### 4.6. Sequence Analysis and Statistical Analysis

Alignments were generated using Clustal Omega [47]. Putative secretory pathway signal peptides (SP) were predicted with TargetP [48] and transmembrane helix predictions were obtained from the Aramemnon database [49,50] with manual adjustments. Arabidopsis sequence data were obtained from the National Center for Biotechnology Information [51] under the following accession numbers: NP_564825.1 (PAM71, At1g64150); NP_193095.2 (CMT1, At4g13590); NP_568535.1 (PML3, At5g36290); NP_173923.1 (PML4, At1g25520); NP_177032.1 (PML5, At1g68650).

Boxplots were generated in Excel 2016 and a one-way ANOVA with post-hoc Tukey HSD test was performed [52].

## 5. Conclusions

The main goal of the present study was to determine the subcellular localization(s) of the three PAM71 homologs: PML3, PML4 and PML5. The localization of PML3 to the Golgi, and of PML4 and PML5 to the endoplasmic reticulum, is clearly different from the chloroplast localizations of PAM71 and CMT1 in a leaf cell. Logically, PAM71 and CMT1 must both be present in chloroplast membranes at the same time. In contrast, the functions of the other three proteins do not necessarily have to be coordinated within individual cells. In agreement with their subcellular localization in the endomembrane system, expression of *PML3*, *PML4* and *PML5* is not restricted to photosynthetic cells, e.g., they are also expressed in non-photosynthetic cells of roots or flowers. The three proteins do not seem to be essential for plant growth and development, however a subtle root growth phenotype was observed in the *pml3* mutant. Overall, root length increased in plants exposed to 500 µM Mn, and this phenomenon is boosted in *pml3*. Taken together, the cellular function of PML3 is distinct from that of PML4 and PML5 and is also evident from the leaf phenotype of the *PML3* over-expressor line. We suspect that PML3 at the Golgi membrane plays a role in balancing excess manganese.

## Supplementary Materials

The following are available online at https://www.mdpi.com/2223-7747/9/2/239/s1, Figure S1: Fluorescence analysis of PML3:GFP, PML4:GFP and PML5:GFP in *N. benthamiana* protoplasts upon leaf infiltration. Figure S2: Selection of stable transformed Arabidopsis lines *Pro35S::PML3:GFP*, *Pro35S::PML4:GFP* and *Pro35S::PML5:GFP*. Figure S3: Analysis of *PML3*, *PML4*, and *PML5* expression. Figure S4: Arabidopsis T-DNA mutant analysis. Figure S5: Analysis of the five genotypes grown under an external supply of 50 nM MnSO4. Table S1: Fragments used for promotor analysis. Table S2: Mean values ± standard deviation of chlorophyll contents of the five genotypes grown under different Mn regimes. Table S3: Mean values ± standard deviation of root length of the five genotypes grown under different Mn regimes. Table S4: Primers used in this study.
