# Supplementary material for: Homologous Proteins of the Manganese Transporter PAM71 Are Localized in the Golgi Apparatus and Endoplasmic Reticulum
Source: Plants (Basel). 2020 Feb 13;9(2):239. doi: 10.3390/plants9020239 (PMC7076475; doi:10.3390/plants9020239)
Supplement: Supplementary file 1 [file plants-09-00239-s001.pdf]

Supplementary Materials

Homologous proteins of the manganese transporter PAM71 are localized in the Golgi apparatus and endoplasmic reticulum

Natalie Hoecker, Anna Honke, Katharina Frey, Dario Leister and Anja Schneider

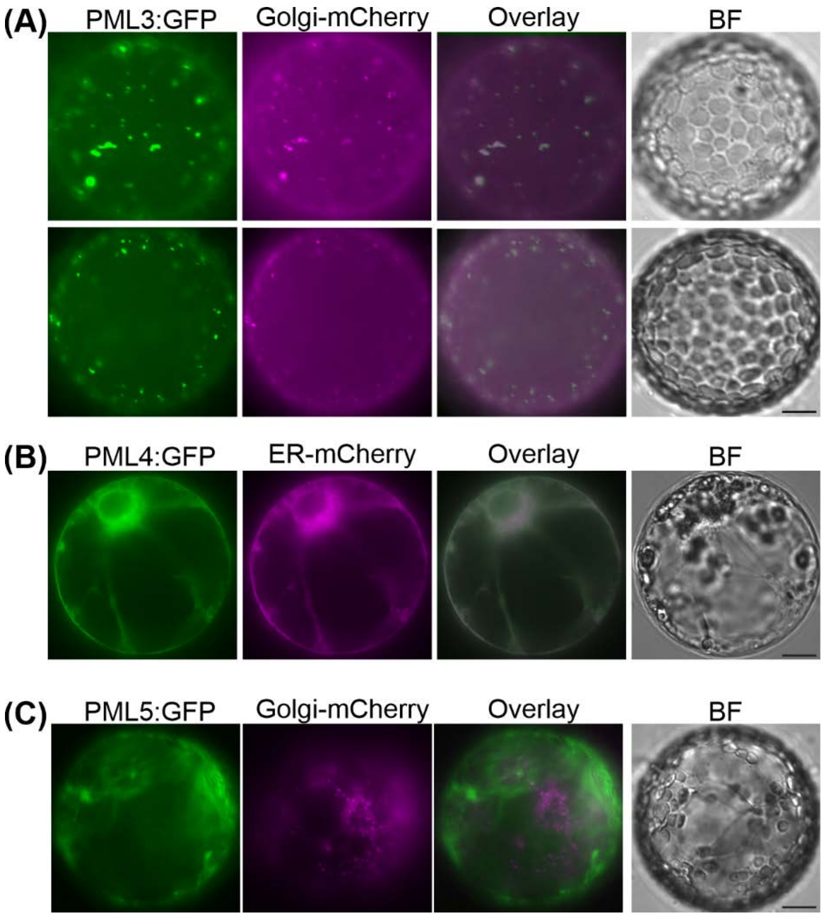

Figure S1. Fluorescence analysis of PML3:GFP, PML4:GFP and PML5:GFP in *N. benthamiana* protoplasts upon leaf infiltration. **(A)** Co-Infiltration with pPro35S::PML3:GFP and pG-rk (= Golgi-mCherry). Upper and lower rows are two different focus layers of the same protoplast. **(B)** Co-Infiltration with pPro35::PML4:GFP and pER-rk (= ER-mCherry). **(C)** Co-Infiltration with pPro35::PML5:GFP and pG-rk (= Golgi-mCherry). The mCherry fluorescence is depicted in magenta, and the GFP fluorescence in green. Overlay pictures were generated and bright field (BF) photographs show intactness of the protoplasts. Scale bar = 10 µm.

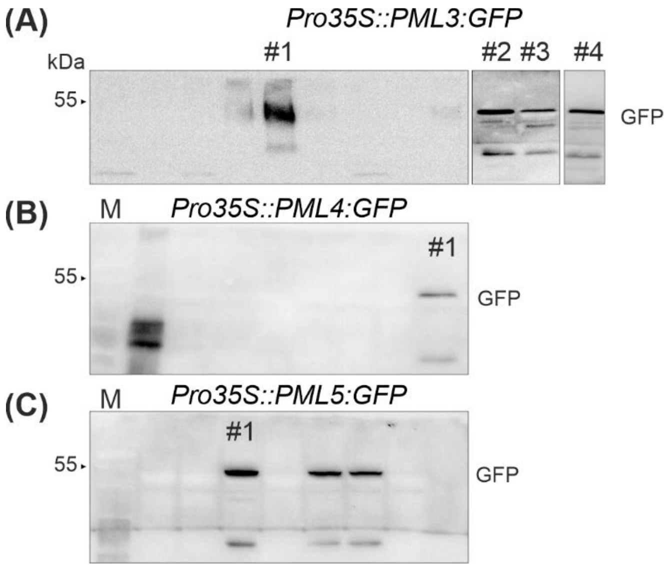

Figure S2. Selection of stable transformed Arabidopsis lines *Pro35S::PML3:GFP*, *Pro35S::PML4:GFP* and *Pro35S::PML5:GFP*. Proteins of individual BASTA resistant plants were isolated, separated by SDS-PAGE and analyzed by immunodetection using the anti-GFP antibody. **(A)** Of nine T1-plants transformed with Pro35::PML3:GFP, one (#1) was selected for microsome isolation (left panel), three further lines with detectable GFP protein were isolated and numbered #2, #3, and #4 (middle and right panel); **(B)** Of eight T1-plants transformed with Pro35::PML4:GFP, one (#1) was selected for microsome isolation; **(C)** Of eight T1-plants transformed with Pro35::PML5:GFP, one (#1) was selected for microsome isolation. M = Marker lane.

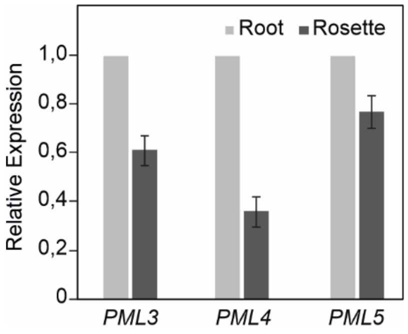

Figure S3. Analysis of *PML3*, *PML4*, and *PML5* expression. Wild-type plants were grown for two weeks under a 12 h/12 h light-dark cycle at 100 μmol photons m^{-2} s^{-1}. The expression levels of the three individual genes in rosette leaves are relative values, based on their expression level in roots. The values were obtained by the $\Delta\Delta C_T$ method: $\Delta C_T = C_T$ ($Gene_{Root}$) - C_T ($Actin_{Root}$) or $\Delta C_T = C_T$ ($Gene_{Rosette}$) – C_T ($Actin_{Rosette}$); $\Delta\Delta C_T$ (Rosette) = ΔC_T ($Gene_{Rosette}$) – ΔC_T ($Gene_{Root}$) and $2^{-\Delta\Delta CT}$ calculated. C_T = Cycle threshold; Mean values (±SD) are based on three biological replicates.

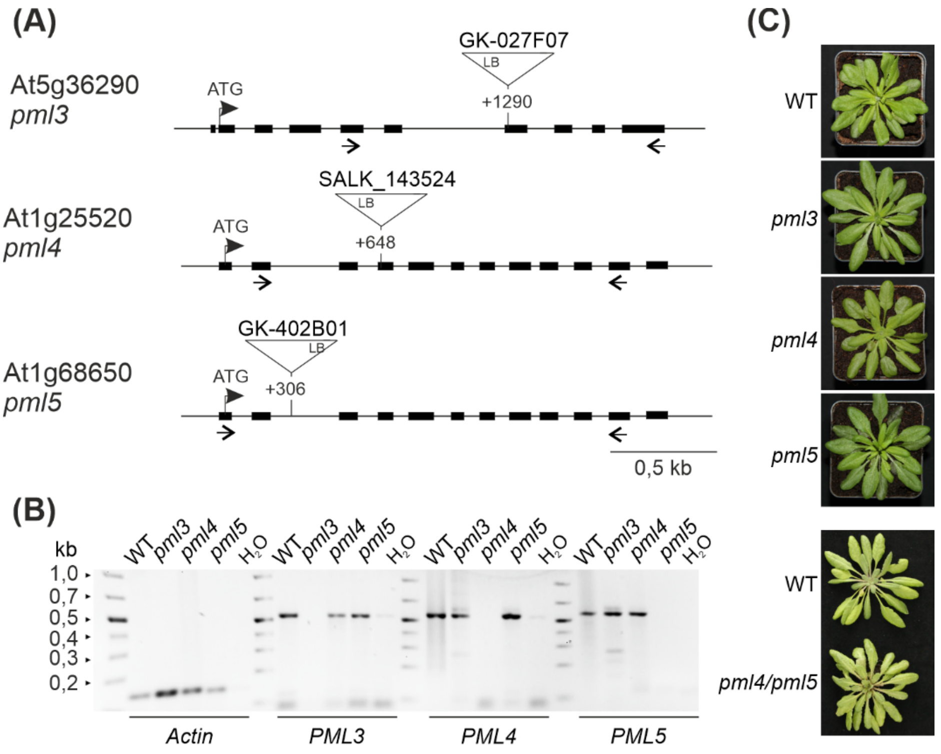

Figure S4. Arabidopsis T-DNA mutant analysis. **(A)** Scheme depicting the location of the T-DNA insertion at the respective gene loci; **(B)** Verification of the knock-out allele status in the respective mutant background (indicated at the top) employing RT-PCR analysis and gene specific primer combinations (*PML3* was amplified with N402563-F and N402563-R; *PML4* was amplified with RT-1g25520-F and RT-1g25520-R; *PML5* was amplified with RT-1g68650-F and RT-1g68650-R). Positions of primers are indicated in (A). As control, *Actin* was amplified using RT-Actin-F and RT-Actin-R primers (for primer sequences see Table S4). **(C)** Greenhouse grown *pml3, pml4* and *pml5* and double mutant plants in comparison to wild-type plants.

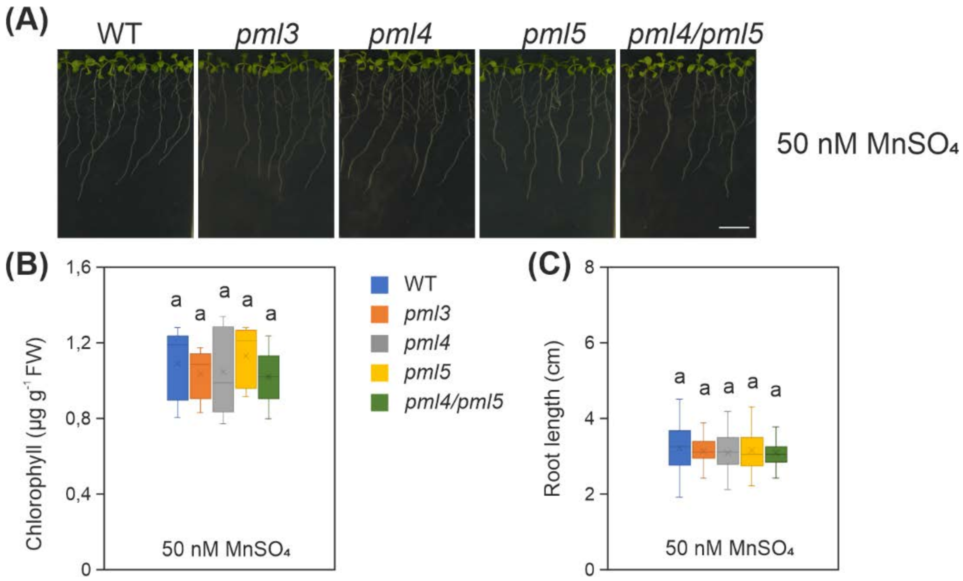

Figure S5. Analysis of the five genotypes grown under an external supply of 50 nM $MnSO_4$. **(A)** Photographs were taken from representative samples. Plants were grown in a vertical position under a 16h/8h light-dark cycle at 100 µmol photons m^{-2} s^{-1} for 10 days. Scale bar = 1 cm **(B)** Five to six plants per genotype and condition were combined for one chlorophyll extraction and at least five extractions were prepared. Data are depicted as boxplots representing the range of values, the exclusive median and the mean, indicated as x ($n \geq 5$). Different letters indicate statistical significance according to ANOVA ($p < 0.05$, Tukey's HSD Test). Mean values and standard deviations are given in Table S2. FW = Fresh weight. **(C)** The length of the primary root of all plants are depicted as boxplots representing the range of values, the exclusive median and mean, indicated as x ($n \geq 35$ per genotype and condition). Outliers are indicated as dots. Different letters indicate statistical significance according to ANOVA ($p < 0.05$, Tukey's HSD Test). Mean values and standard deviations are given in Table S3. Poorly-germinated seeds were excluded from the analysis.

Table S1. Fragments used for promotor analysis.

Gene	Sequence (5′ - 3′)	Fragment Size
PML3	tgcatcacttggagttagatttgttttaaagaaattattatataaacaaatttataatatttttttctgc<br>gatatcacgaagggtttgagttctagtttaattaaaataattcttattaaattaaattaaaataaattaa<br>attcaaataacataaactaattatagtcaaattatatttgataaaactaaaataaataaatatattaatt<br>tatttttctcatgatgtatgatatatatgatttacattttgattttaaaatataaattagcaccaaaaat<br>aattaattttaaaaaaatatagcgactgtttatagcgacattcaaactaacaacacagcgacaattatt<br>agtgattatcgctgtaattggtcgctgagtccagcgacagacaaacgaacaatatatgttgtcgctggtt<br>gttgtcgctggtcgctgactaagtttgagaaaatatattttaatgaaaaagatatattttagtggtattt<br>ttgagaaaatatatattctagttttgactaagtttgatgcaaatactgagttagaagtcgtgataagatt<br>tgagggaatgcttagcaaacaatcgatattgataaaaacaaaaattgcttcaaacattttttatttccctc<br>ttgatgtcatgacattcctcctaaagaacctccaaaatagagtctaaaaatcttctctttttgctttgga<br>tccacttcgtctaataaaaaaacaacaagtatggagtctcaatcgttgaacctccacgagattcctctca<br>ctcgacatccttatttgctattgttcatggtttaattacctgaatactttacaaaaaactgaaaatatcg<br>aaccggaatcaaaacaaatcgagaaacccaaaaccaacacaagtagtattttttttttttttttaaagta<br>gtcttttgtaaaaatttataatattgatgttaaaatagtattttgtaaaaatttataatattgatgttca<br>cgataaaaaaaattagtttttcaaatcttttttttagtatatagtgcaatttcttaaaatatagtagcaact<br>agtaaaatctggaatatataataagatttaaaaatatttttgtaaaccgtgttaatttgattaattccat<br>tacatcttcaaaaccgtacagatctctgtttcggagcagcttcttcacacgctcaagctcaccgatcgat<br>tcttccaatgctcttctaattcgtaagtaattttccagatccaacctttctcagattcgctctttcccga<br>gaaaatttgaagaaacagagattcatcatttctcttcctctcaaaaatcagatctgcaaaactctcaga<br>tcactc[ATG]	-1021 bp to +315 bp with respect to transcription initiation site
PML4	cggttagtgtggagattgttgttttagattttattaagtcagccacttttgagtgtttcacatcttcgca<br>ctttgacaaatttttatttggctatgagcttagctttgttaatgatgactttcattcaaattataacagct<br>cacttatttcattttatccagacctatgtctataaagctatatgatactaaacaattaacctaaaaagaa<br>agctataactgtaaaccaatacatacatacgcaatctgacaacaacaaaaaaacatacatatgcatatca<br>tgttaaggtattaacaatgtggaggaatttgctgacttaaaaatggtgaatttatggtccctcatgggcc<br>tgtgggtaactcttatctaggcttttaacttggatgtttgggccatttcataccccaagtctactacatt<br>aactcattaaccttgctgcctaaatctaaactataaattctaaaatacacttctaatctcagcttaacat<br>gatatgcagttgagatagatagaatattgccacctgattttagttattcatcaattttagaattaaataa<br>attaatattaatataagtacagttggatcaaatcaaatataattacatacgtttgcgaatcaattcgcca<br>ccaatcagaaatcggtcaatttttttttatttgtataaaatagtaggataaaacgaaataaaatttagtt<br>gagataagcgaaaaatactaaaatacccaatcagttttgagcatctctcaaaaacttacgtgttatacta<br>ttctaaacgttacgtctcaatcgtgatcgcgacaagttagttatcaaagaagtaaaagtctttattgttt<br>tgctcgagcaagtgacgcaagtcgcaaacaacaacacaagggaacaaattaacatcttctaaactcaagt<br>tgggttttctgggtcccacacattaaaatcgttggttgttatcaatccacgtggtaagatcttaagggtt<br>aaagaatcagaatcaaattcacctacaatccccaaaaaaaaaggattcatcttttagctcttcagcgaat<br>gttcgtctcgtctatttgctctctctcgacgactgttaattaactccggcgattgatcactccgtcttct<br>ttgtaacacagccggagaaaaatcgaaagtttcgtttttttcttctccttcgtagctaattcgccgtcga<br>ttttataattcggcagcc[ATG]	-971 bp to + 237 bp with respect to transcription initiation site
PML5	ccaataatgaaattggttttggacaagagagtagtagtacatttggcagagtcaatgacgtctaggctct<br>gtttagtcttgtgggtattttcttgtttttttactcgtcagaatccgatcttgtctctttcttctcttctc<br>atttctttttgaaacatttgggagaagtaaataatctttagaacatttctctgcatcatatgtaccattt<br>tttttctttcataaataaaaaggtttagagtcttgagtgtttctcttttccgaagataattagatacagt<br>acattagattgttacatttgaccaaaacaaatcttctacaacttcctattgatgatgatttgacatgtgg<br>aggaataaagaaagcaatgttgttgttgttgttacacagttttaatcatttagctgtgcgagcatagaga<br>ctattagccattacttataagccaacacaaaatttacttggtcttagagctttggattcagtaggtgcaa<br>aaggtcttaccgggactattcccaacaacatttctgtagatcttactttttataactctgtcagataatt<br>agatatacttgtacgaaagtttactatatgaaataaacaagatcatgtagttctcactggttaataactg<br>tttaactatgagtctagaagaaagctcgatataaactgtttcatgtaactttgaggtactttttttttc<br>atatgtgtttgtttggccaaaacagtttaacactttcaatactcgaagctacttaggtaatcctcttcta<br>tgtggccattgttaatcataaattctttgttgtcctgctttgtgtaacaaaaccataacataactcatgt<br>tggttcctttgtacctttgtctttgaagaaatgtgatgtctcaactctcaaaaatacgaattgtgtggta<br>tagaattcttcttgtctcctaaagttggtaatagagcaacaatggaacaataataaatgaacacaacact<br>ctctatgactcaagtcaactgtcaaagcttggttccctgggtcctacataacaaaatcttcgaatcatat<br>tcttccacgtggcaagatctaaccaatgagaatctatttttccatatgctctccacactcccaccaaaca<br>aatcccaagaatcaacaaaagaaaaaacattcaccttctcttacgcgactctcgccggaaacagatcgct<br>ccgccgtgaactcggaagcc[ATG]	-1116 bp to + 94 bp with respect to transcription initiation site

Sequences in red denote the 5′ UTR regions, sequences in blue denote an intron region within the 5′UTR, the ATG denoted as [ATG] is not included in the amplified fragment, for primer sequences see Table S4. Annotations are based on the 21st public release of TAIR@Phoenix data (01. Jan 2020) at https://www.arabidopsis.org/

Table S2. Mean values ± standard deviation of chlorophyll contents of the five genotypes grown under different Mn regimes.

	50 nM $MnSO_4$	5 µM $MnSO_4$	50 µM $MnSO_4$	500 µM $MnSO_4$
WT	1,12 ± 0,21	1,08 ± 0,23	1,03 ± 0,31	0,60 ± 0,18
pml3	1,06 ± 0,15	1,03 ± 0,21	1,06 ± 0,30	0,64 ± 0,07
pml4	1,07 ± 0,26	1,09 ± 0,17	0,92 ± 0,14	0,75 ± 0,29
pml5	1,17 ± 0,18	1,03 ± 0,16	0,99 ± 0,10	0,72 ± 0,22
pml4 pml5	1,04 ± 0,20	1,05 ± 0,16	0,92 ± 0,13	0,78 ± 0,22

Five to six plants per genotype and condition were combined for one chlorophyll extraction and at least 5 chlorophyll extractions were prepared (n ≥ 5). Chlorophyll content is given in (µg mg^{-1} FW). FW = Fresh weight.

Table S3. Mean values ± standard deviation of root length of the five genotypes grown under different Mn regimes.

	50 nM $MnSO_4$	5 µM $MnSO_4$	50 µM $MnSO_4$	500 µM $MnSO_4$
WT	3,26 ± 0,67	3,34 ± 0,55	3,37 ± 0,54	3,74 ± 0,60
pml3	3,18 ± 0,38	3,39 ± 0,29	3,45 ± 0,72	4,60 ± 0,57
pml4	3,14 ± 0,55	3,24 ± 0,52	3,54 ± 0,71	3,59 ± 0,66
pml5	3,19 ± 0,49	3,40 ± 0,50	3,57 ± 0,60	3,99 ± 0,65
pml4 pml5	3,14 ± 0,30	3,11 ± 0,54	3,18 ± 0,47	3,68 ± 0,50

Root length was determined from at least 35 individual plants per genotype and condition. Root length is given in (cm).

Table S4. Primers used in this study. Sequences required for cloning are underlined.

Primer name	Sequence (5′-3′)	Application
N402563-F	CAT TGA TGG CTA TGC GAC ATC CTA	Genotyping
N402563-R	CGA GGA AGA GTA AGC CTC CAA CAG	
N664220-F	ATC TTT TAG CTC TTC AGC GAA TGT	
N664220-R	AAC CCC TGC CAA CAA AGT CAG ATA	
N438509-F	AAC AAA ACC ATA ACA TAA CTC ATG	
N438509-R	AAA ACA TAT ACC GAA GCT GAG GAG	
GK-LB	ATA TTG ACC ATC ATA CTC ATT GC	
Salk_LB	GTC CGC AAT GTG TTA TTA AGT TGT C	
35S-F	GCA AGA CCC TTC CTC TAT ATA AG	
GFP-R	CTT CAG GGT CAG CTT GCC GTA	
N402563-F	CAT TGA TGG CTA TGC GAC ATC CTA	RT-PCR
N402563-R	CGA GGA AGA GTA AGC CTC CAA CAG	
RT-1g25520-F	GCT ATG ACC TTT GTA TCG GAG ATT	
RT-1g25520-R	ACA GCA GCA GTG GTG CAC AAA AAC	
RT-1g68650-F	GGT CAC TTT TGC AGG GTT TTA CTA	
RT-1g68650-R	ACA GCA GCT GTG GTA CAC AAT GTT T	
RT-Actin-F	GCA ACT GGG ATG ATA TGG AAA AGA	
RT-Actin-R	CAA AGG AGG GCT GGA ACA AGA CT	
ProPML3-F	CAC CTG CAT CAC TTG GAG TTA GAT TTG T	Construction Promotor-GUS Lines
ProPML3-R	GAG TGA TCT GAG AGT TTT GCA GAT	
ProPML4-F	CAC CCG GTT AGT GTG GAG ATT GTT GTT T	
ProPML4-R	GGC TGC CGA ATT ATA AAA TCG ACG	
ProPML5-F	CAC CCC AAT AAT GAA ATT GGT TTT GGA C	
ProPML5-R	GGC TTC CGA GTT CAC GGC	
GA-PML3-F	AAT TTA CTA TTC TAG TCG AAT GGG TTT GAT TTC AAA CC	Construction GFP Lines Transient & Stable Expression
GA-PML3-R	CCT TGC TCA CCA TCA ATG GAG GAT AGA AAT AGG	
GA-pB7FWG2-F	ATG GTG AGC AAG GGC GAG	
GA-p35S-R	TCG ACT AGA ATA GTA AAT TGT AAT GTT GTT TG	
PML4-F	CAC CAT GAG CTC GGT TTT GCA G	
PML4-R	AGC CTC AAC AGA AGT AAG ATA CGA TTG GAT	
PML5-F	CAC CAT GGG GTC ACT TTT GCA GGG TTT TAC TAA A	
PML5-R	AGC ATC AAC CGG AGT AAG AAG CGA ATG AAT	
